# Mammary‐type myofibroblastoma of the perineum: Typical or rare location?

**DOI:** 10.1002/iju5.12423

**Published:** 2022-02-23

**Authors:** Akihiro Naito, Yuta Takeshima, Sayuri Takahashi

**Affiliations:** ^1^ Department of Urology IMSUT Hospital The Institute of Medical Science The University of Tokyo Tokyo Japan; ^2^ Division of Innovative Cancer Therapy Advanced Research Center The Institute of Medical Science The University of Tokyo Tokyo Japan

**Keywords:** mammary ridge, myofibroblastoma, perineum, subcutaneous tumor

## Abstract

**Introduction:**

Mammary‐type myofibroblastoma is a rare benign tumor, mainly arising along the embryonal mammary ridge. We report a rare case of mammary‐type myofibroblastoma of the perineum.

**Case presentation:**

A 37‐year‐old Japanese man presented with a 20 mm, progressively‐growing painless mass in the right perineum. Computed tomography showed a subcutaneous tumor with a strong contrast effect. Upon total resection, pathology showed a spindle‐cell tumor positive for desmin but negative for CD34. Further immunohistochemistry showed loss of Rb expression, leading to differential diagnosis. We could not evaluate the exact rarity of the perineal location due to categorization in past reports.

**Conclusion:**

Due to the similarities between mammary and anogenital tissue, we suggest that tallying perineal and vulvar areas separately from the embryonic mammary ridge sites may be beneficial in gaining insight into the pathophysiology of this tumor.

**Abbreviations**
MLGs: mammary‐like glands
MTMF: mammary‐type myofibroblastoma

**Keynote message**

We present one case of mammary‐type myofibroblastoma of the perineum. Past reports lead us to believe it would be beneficial to tally perineal and vulvar tumors of this type separately from conventional mammary ridge sites.

## Introduction

Mammary myofibroblastoma is an uncommon benign breast tumor, composed of spindle cells in tight fascicles with myofibroblastic differentiation. Mammary myofibroblastoma was first reported in 1981 as four cases of a “benign spindle cell tumor of the breast” and defined as a distinct entity in 1987.^1^, ^2^ In 2001, McMenamin et al. reported nine cases of extra‐mammary soft‐tissue tumors histologically identical to mammary myofibroblastoma and coined the term “MTMF”.^3^ Distribution of MTMF have led to the hypothesis that these tumors arise along the embryonic mammary ridges from the axilla to mid‐groin.^3^, ^4^ However, MTMF has since been reported in several locations distant from the mammary ridge, such as the liver, seminal vesicle, scrotal sac, abdominal wall, popliteal fossa, and toe.^5^, ^6^, ^7^, ^8^, ^9^, ^10^ We herein present a case of MTMF arising in the perineum, and review the literature with an emphasis on anatomical location and its possible implication in an embryological setting.

## Case presentation

**Fig. 1:**
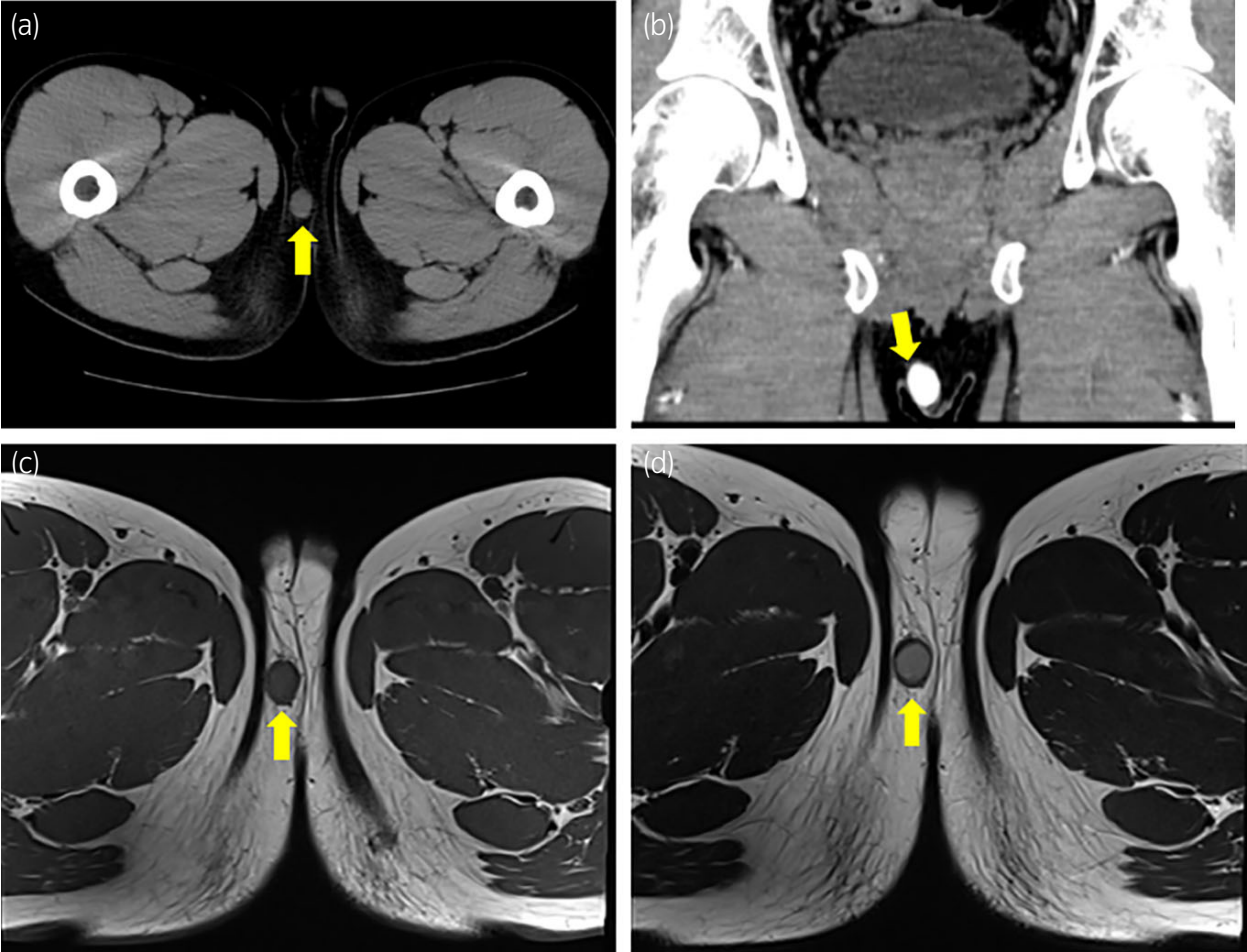
Radiological findings of perineal tumor. (a) CT shows a 20 mm subcutaneous tumor in the right side of the perineum. (b) Contrast ‐enhanced CT shows strong enhancement. (c) T1‐weighted MRI shows a well‐circumscribed mass with a mild hyperintensity. (d) T2‐weighted MRI shows a hyper‐intense mass

A 37‐year‐old Japanese man presented with a progressively enlarging swelling in the right side of the perineum. At diagnosis, the mass was 20 mm in diameter, fully mobile, well‐circumscribed, and painless. There was no past medical history, and all laboratory data were within normal limits. Contrast‐enhanced computed tomography showed a 20 mm soft‐tissue lesion with a strong contrast effect (Fig. 1a,b). T1/T2‐weighted magnetic resonance imaging showed a hyperintense mass with gadolinium enhancement (Fig. 1c,d). Based on these findings, possibility of malignancy was considered low. Total resection of the tumor was performed without complications.

**Fig. 2:**
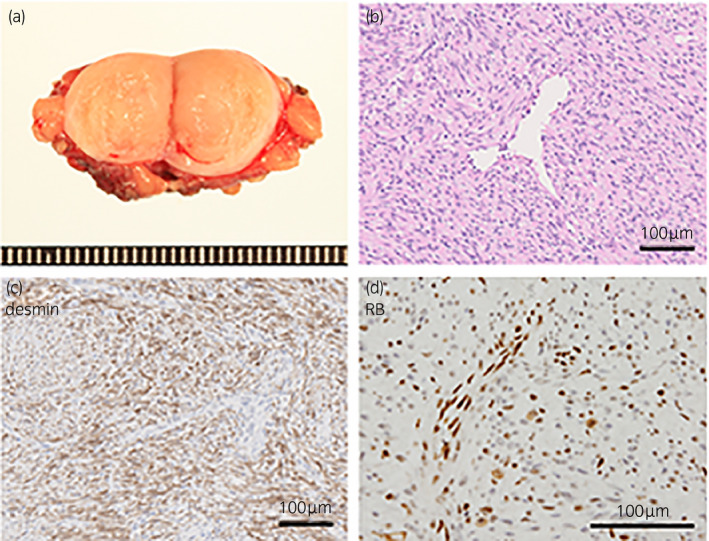
Pathological findings of perineal tumor. (a) Tumor excision reveals a smooth, yellow‐tan tumor. (b) Bland spindle cells seen on hematoxylin‐eosin staining. (c) Spindle cells diffusely‐positive for desmin staining. (d) Strongly‐attenuated Rb positivity in spindle cells. Positive cells seen here are comprised of vascular endothelial cells and lymphocytes

The cut surface of the mass revealed a smooth, yellow‐tan tumor with no hemorrhaging or necrosis (Fig. 2a). Histopathology showed a spindle cell tumor with little atypia (Fig. 2b). The spindle cells showed diffuse immunohistochemical labeling with desmin, but were negative for CD34, α‐smooth muscle actin, estrogen receptor and progesterone receptor staining (Fig. 2c). Staining for Rb protein revealed a loss of Rb expression in the spindle cells (Fig. 2d). The resection margin was negative for tumor cells, and postoperative follow‐up shows no recurrence of the tumor at 6 months.

## Discussion

MTMF is a rare benign tumor occurring outside of the breast. The majority of patients in past reports were men, with most patients aged 41–80.^4^, ^5^, ^6^, ^7^, ^8^, ^9^, ^10^ MTMF frequently contains a fatty component visible on CT and MRI, but our case contained little fat, leading to difficulty in pre‐operative differentiation from other hypervascularized tumors such as paraganglioma or angiofibroma.^6^, ^11^ There is only one report of recurrence following incomplete resection, no reports of recurrence after complete resection, and no reports of post‐operative morbidity.^9^

Pathological diagnosis is accomplished chiefly by morphological findings, that is, bland, uniform spindle cells in short fascicles mixed with bands of thick hyalinized collagen and varying amounts of fat. However, differentiation from tumors of similar morphological composition such as spindle cell lipoma or angiomyofibroma may be difficult, and immunohistochemical staining is often beneficial (Table. 1). MTMF is typically positive for CD34 and desmin, with percentages reported as 89% and 91%, respectively.^4^ The desmin positivity aided in differentiation from spindle cell lipoma. CD34 is involved in angiogenesis, but our case was negative for CD34 despite showing hypervascularity. Estrogen and/or progesterone receptor expression has been reported in three previous case reports, but was negative in our case.^5^, ^7^, ^8^ MTMF, along with spindle cell lipoma, cellular angiofibroma, and vaginal myofibroblastoma, compose a group of tumors (the “13q/RB1 family”) in which 13q14 chromosomal alteration leads to loss of Rb expression.^10^, ^12^, ^13^ This loss of nuclear Rb expression in MTMF cells has been reported in 92% of cases.^4^ In this case, desmin positivity and loss of Rb expression were beneficial in reinforcing our diagnosis.

**Table 1:** Pathological differentiation of MTMF from similar subcutaneous tumors

Type of tumor	Characteristics different from MTMF
Spindle cell lipoma	Fat component usually predominant
	Normally negative for myogenic markers (desmin, CD34)
Cellular angiofibroma	Thicker hyalinized vessels
	Lacks thick collagen bundles
	Normally negative for myogenic markers (desmin, CD34)
Angiomyofibroblastoma	Alternating zones of hypo and hypercellularity
	Normally CD34 negative
	No loss of Rb1(13q14)
Solitary fibrous tumor	STAT6 positive
	No loss of Rb1(13q14)
Pseudoangiomatous stromal hyperplasia	Usually not mass forming
	No loss of Rb1(13q14)
Nodular fasciitis	Loose storiform proliferation of spindle cells
	Inflammatory cells (lymphocytes, histiocytes) present
	CD34 and desmin negative
	MYH9‐USP6 fusion confirmed by FISH or PCR
Fibromatosis	Long, sweeping fascicles, infiltrative appearance
	CD34 negative, beta‐catenin positive
	CTNNB1 mutation
Metaplastic spindle cell carcinoma	More atypia, mitoses, and infiltrative borders
	CD34 negative, cytokeratin, and p63 positive
Invasive lobular carcinoma	More atypia, mitoses
	CD34 and desmin negative, cytokeratin positive

Concerning location, the largest case series to date classifies 65 cases (45%) in the “inguinal, vulvar, perineal, and scrotal regions” with no further details.^4^ Although we presume a large portion of these 65 cases were located in the most common inguinal area, and few in the perineum of which there are no other reports, we cannot know exactly how rare perineal MTMF is. It was hypothesized that these tumors occur along the embryonic mammary ridge from the axilla to the groin.^3^, ^4^ The basis for this is that ectopic breast tissue is often seen along the mammary ridge, and that tumors similar to those seen in breast tissue such as fibroadenoma, intraductal papilloma, and adenocarcinoma often arise from these tissue..^14^, ^15^, ^16^It is suggested that myofibroblastic tissue along the mammary ridge may similarly be predisposed to form MTMF. The dilemma we faced was that past reports disagreed on whether the perineum is included the mammary ridge.

Interestingly, we found that the male perineum is often discussed in the same manner as the vulva.^17^ This is understandable from an embryological standpoint in that the male perineum and female vulva both derive from the vestibular plate. After canalization of the vestibular plate forms a groove, an androgen‐dependent mechanism forms the perineum and urethra in males, while the groove remains open forming the labia minora in females.^18^, ^19^ This analogy in the development of the male perineum and female vulva may lead to similarities in pathology. Regarding tumors of the vulva, van der Putte detailed “MLGs” found in abundance in the anogenital region of both males and females, named for their morphological similarity, but not identicality, to mammary glands.^20^ He theorized that the development of MLGs are so widely separated by time and space from the mammary ridge that they were of different origin. Furthermore, many tumors usually found in the breast have since been attributed to vulval MLGs, such as hidroadenoma papilliferum, apocrine cystadenoma, and adenocarcinoma, despite the anatomical distance from the pectoral region. Although there is no direct evidence linking MTMFs to MLGs, we found it interesting that there exists an abundance of evidence on similarity between the mammary and anogenital regions independent of the popularized mammary ridge theory. These evidence lead us to believe that it may be beneficial to count vulvar and perineal MTMF separately from those on the mammary ridge from the axilla to the groin. Further accumulation of data on the frequency of MTMF at this location may shed light on the relation between the vulva or perineum and the mammary ridge.

## Author Contributions

Akihiro Naito: Conceptualization; Formal analysis; Visualization; Writing – original draft. Yuta Takeshima: Data curation; Formal analysis; Investigation; Writing – original draft; Writing – review & editing. Sayuri Takahashi: Conceptualization; Methodology; Project administration; Supervision; Writing – review & editing.

## Conflict of interest

The authors declare no conflicts of interest.

## Approval of the research protocol by an Institutional Reviewer Board

Not applicable.

## Informed consent

Obtained from patient.

## Registry and the registration no. of the study/trial

Not applicable.

## Funding source

This study was not funded by any institution.
